# Atypical Multibacterial Granulomatous Myositis in a Horse: First Report in Italy

**DOI:** 10.3390/vetsci7020047

**Published:** 2020-04-21

**Authors:** Claudia Rifici, Anna-Rita Attili, Davide De Biase, Roselane Gonçalves dos Santos, Núbia Seyffert, Thiago Luiz De Paula Castro, Henrique Cesar Pereira Figueiredo, Carmelo Scaramozzino, Stefano Reale, Orlando Paciello, Vincenzo Cuteri, Sharon Jane Spier, Vasco Azevedo, Giuseppe Mazzullo

**Affiliations:** 1 Department of Veterinary Science, University of Messina, Polo Universitario dell’Annunziata, 98168 Messina (ME), Italy; 2 School of Biosciences and Veterinary Medicine, University of Camerino, Via Circonvallazione 93/95, 62024 Matelica (MC), Italy; 3 Department of Pathology and Animal Health, University of Naples “Federico II”, Via Federico Delpino 1, 80137 Napoli, Italy; 4 Institute of Biological Sciences, Federal University of Minas Gerais, Belo Horizonte, Minas Gerais 31270-901, Brazil; 5 Institute of Biology, Federal University of Bahia, Salvador-Bahia 40170-115, Brazil; 6 Institute of Health Sciences, Federal University of Bahia, Salvador-Bahia 40170-115, Brazil; 7 School of Veterinary, Federal University of Minas Gerais, Belo Horizonte, Minas Gerais 31270-901, Brazil; 8 Scaravet Equine Breeding Center, Via Don Minzoni, 29, 89124 Reggio Calabria, Italy; 9 Molecular Biology Department, Experimental Zooprophylactic Institute (IZS) of Sicily, Via Gino Marinuzzi 3, 90129 Palermo (PA), Italy; 10 Department of Veterinary Medicine and Epidemiology, University of California, Davis, CA 95616, USA

**Keywords:** bacterial myositis, horses, *Corynebacterium pseudotuberculosis*, *Glutamicibacter creatinolyticus*, *Dietzia* spp.

## Abstract

Infectious causes of myositis are reported relatively uncommonly in horses. Among them, bacterial causes include *Streptococcus equi* subsp. *zooepidemicus*, *Actinobacillus equuli*, *Fusobacterium* spp. *Staphylococcus* spp, and *Corynebacterium pseudotuberculosis*. Infection can be spread to muscles via haematogenous or extension from skin lesions. Parasitic myositis has also been documented. In this report, a 12 year-old Italian Quarter Horse mare presented with diffuse subcutaneous nodules and masses ranging from 2 × 3 to 5 × 20 cm in size, and adherent to subcutis and muscles that were first macroscopically and cytologically diagnosed as pyogranulomas. Subsequently, histological, molecular, bacteriological, and biochemical investigations were performed. All the data obtained allowed to diagnose a severe and diffuse multibacterial granulomatous myositis caused by *Corynebacterium pseudotuberculosis* and *Corynebacterium amycolatum*. Following the therapy and an initial disappearance of most of the lesions together with a general improvement of the mare, the clinical condition deteriorated, and new nodules appeared. Matrix-assisted laser desorption/ionization time-of-flight (MALDI-TOF) and PCR techniques revealed the presence of bacteria as *Glutamicibacter creatinolyticus* and *Dietzia* spp. To the authors’ knowledge, this case report represents the first description of multibacterial granulomatous myositis due to *Corynebacterium pseudotuberculosis*, *Corynebacterium amycolatum*, *Glutamicibacter creatinolyticus*, and *Dietzia* spp. in a horse reared in Italy.

## 1. Introduction

Myopathies are disorders affecting skeletal muscle and, in adult horses, include nutritional myopathies, infectious diseases, immune-mediated disorders, toxic myopathy, inherited myopathies, endocrinopathies, pasture-associated rhabdomyolysis, malignant hyperthermia, and exertional rhabdomyolysis [1]. Granulomatous myositis is a rare disorder characterized by non-caseating granulomatous inflammation of the skeletal muscles, which can present as either an idiopathic entity or accompanying several conditions, such as sarcoidosis and other inflammatory and infectious diseases [2,3]. Less commonly, infectious agents, such as bacteria, viruses, protozoa, and parasites could be involved. Bacterial infection, occurring in association with trauma, is the most common form of primary infectious myositis in horses. Causes of bacterial myositis include *Streptococcus equi* subsp. *zooepidemicus*, *Actinobacillus equuli*, *Fusobacterium* spp., and *Corynebacterium pseudotuberculosis* that result from haematogenous spreading and/or lesions of the skin [4]. *Corynebacterium pseudotuberculosis* is a Gram-positive, pleomorphic, facultative intracellular pathogenic bacterium belonging to the *Corynebacterium*, *Mycobacterium*, *Nocardia*, and *Rhodococcus* (CMNR) group of the *Actinobacteria* Phylum, one of the largest lineages of bacteria according to molecular and phylogenetic analyses based on 16s rDNA. *Corynebacterium pseudotuberculosis* is an important animal pathogen and it is the etiological agent of a disease that is commonly called Caseous Lymphadenitis (CL) or cheesy gland in sheep and goats [5]. While vaccination against CL may reduce the prevalence of infection [6], the disease is present in all the world’s major sheep and goat production areas, causing significant economic losses by affecting wool, meat, and milk production [7,8,9,10,11]. *Corynebacterium pseudotuberculosis* has also been isolated from other animal species such as cattle, camels, swine, buffaloes, and horses, in which it causes Ulcerative Lymphangitis and Pigeon Fever [7,12,13,14]. In horses, the disease occurs as ulcerative lymphangitis, caused by the transmission of bacteria from insect vectors or contamination of skin abrasions. Abscesses in horses are also commonly seen in the pectoral region and ventral midline, which are common sites for feeding of insects such as horn flies, house flies and stable flies.

*Corynebacerium pseudotuberculosis* can rarely affect humans, causing lymphadenitis, and contamination occurs through contact with infected animals [5,12,15]. 

The aim of this study was to describe the pathological and microbiological aspects of a multibacterial granulomatous mysositis diagnosed in a horse reared in Italy.

## 2. Case Description 

**Figure 1:**
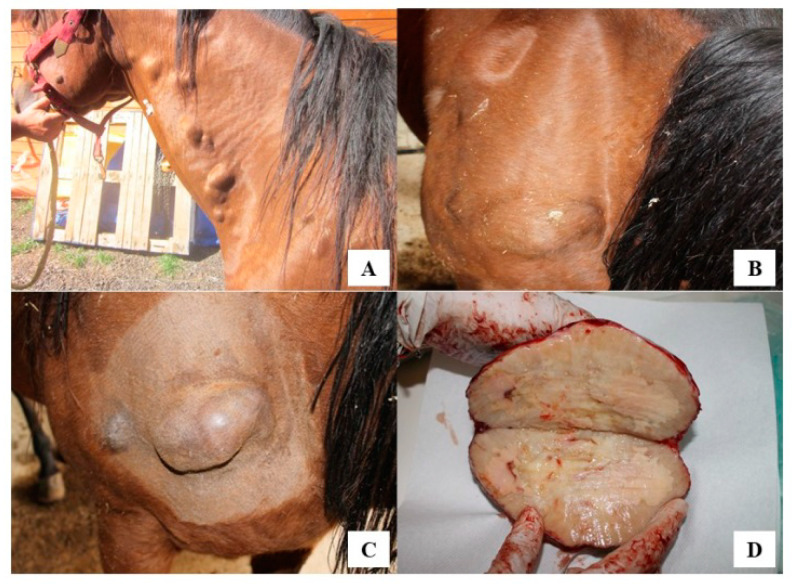
(**A**) Several single or multiple subcutaneous nodules and masses, from 3 to 20 cm in size. (**B**) Nodules mainly localized at shoulder regions. (**C**) Nodules adherent to muscles, painless and firm in consistency. (**D**) On cut section, evidence of necrotic caseous material surrounded by a fibrotic capsule with small purulent-like foci externally to the necrotic tissue.

A 12year-old Quarter Horse mare, born and housed in southern Italy (Reggio Calabria) as a companion animal was presented because of diffuse subcutaneous nodules and masses. Lesions ranged from 2 × 3 (Figure 1A) to 5 × 20 cm (Figure 1B) and were mainly localized at shoulder and neck regions. Through cytology, a pyogranulomatous condition was diagnosed and a first treatment with penicillin (10,000 IU/kg) and dihydrostreptomycin (7.5 mg/kg) intramuscularly, once daily, for two weeks was given but without any improvement or disappearance of lesions [16]. Due to worsening of the clinical condition over this period that included weight loss, lethargy, and ocular discharge, 3 masses were surgically removed and a third-generation cephalosporin (Ceftiofur: 2.2 mg/kg b.w.) and tetracycline (5 mg/kg intravenously once daily) were administered for 10 consecutive days. The excised masses were sent to the Unit of Pathology, Department of Veterinary Sciences, University of Messina (Italy), for histological examination. Macroscopically, the excised masses appeared well-vascularized and adherent to the subcutaneous tissues and underlying muscles. On cross section, fibrous connective tissue surrounded a caseous and necrotic material with dispersed small multiple purulent-like foci (i.e., pyogranulomas) (Figure 1C,D). 

From different subcutaneous nodules, four aliquots of excised tissues were sampled: one was fixed in formalin (10%) and embedded in paraffin for histological diagnosis, one aliquot was subjected to molecular investigation, and two aliquots were frozen at −20 °C for further analysis. Briefly, tissue sections of 3–4 μm were stained with Hematoxylin-Eosin (HE), Masson Trichrome (Code 04-011802, Bio-Optica, Milan, Italy), Periodic acid—Schiff (PAS) (code no. 04-130802, Bio-Optica, Milan, Italy), methenamine silver (Grocott) (code no. 04-043823, Bio-Optica, Milan, Italy) for the histological identification of fungi, and Gram (code no. 04-1008002, Bio-Optica, Milan, Italy) for the histological identification and differentiation of bacteria.

**Figure 2:**
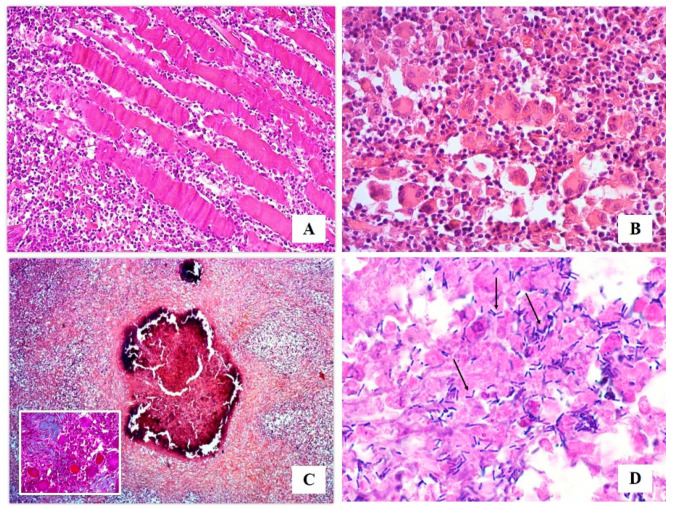
Histological examination: (**A**) widespread inflammatory infiltrate invading the endomysium (HE, 10× magnification); (**B**) mixed inflammatory infiltrate constituted by granulocytes neutrophils and eosinophils, macrophages, lymphocytes, plasma cells, epithelioid and multinucleated giant cells (HE, 40× magnification); (**C**) collagenolytic degeneration areas (Masson Trichrome, 20×), and foci of necrosis associated with calcification (HE, 5× magnification); (**D**) Gram stain allowed to detect small aggregates of rod-shaped, straight or slightly curved blue Gram-positive microorganisms showing an irregular swelling at one or both end (“Club Shaped”), and grouped together in a characteristic way often forming a “V” or a “L” (arrows) (Gram, 100× magnification).

By histological examination, a diffuse mixed inflammatory infiltrate, consisting of granulocytes neutrophils and mainly eosinophils, macrophages, lymphocytes, plasma cells, as well as epithelioid and multinucleated giant cells (Figure 2A,B), was present in the endomysium. This infiltrate was often arranged between collagenolytic degeneration areas, evidenced by Masson Trichrome staining, or around foci of necrosis associated with calcification (Figure 2C). PAS and Grocott stains revealed no fungal presence. Gram staining allowed to detect small aggregates of pleomorphic, rod-shaped Gram-positive bacteria both inside macrophages or free, mostly in caseous necrotic areas. Bacteria had an irregular swelling at one or both end (“club shaped”), they were straight or slightly curved and grouped together in a characteristic way often forming a “V” or a “L” (Figure 2D).

On the basis of these results, a diagnosis of bacterial granulomatous myositis was made [2,3]. Hence, in order to reveal the etiologic agent, cause of the pathology, an aliquot of tissue was sent to the Experimental Zooprophylactic Institute (IZS) of Sicily, Palermo, Italy. The DNA was isolated and analyzed via PCR targeted to the ribosomal region and sequences were amplified by primers annealing at 16S of ribosomal genes, specific primers to amplify a species-specific DNA region of *Corynebacterium* spp. [17]. Amplification of the 16S rRNA region was performed by using pA 5′-AGAGTTTGATCCTGGCTCAG and pH 5′-AAGGAGGTGATCCAGCCGCA universal primers [18]. The full 16S rRNA region was amplified by PCR in a final reaction volume of 50 μL. Each reaction mixture contained approximately 10 ng of template DNA; 0.4 pmol (each) forward (pA) and reverse (pH) primers; 10 μM (each) dATP, dCTP, dGTP, and dTTP; 10× reaction buffer containing 1.5 mM MgCl2 (AB) and 2 U of Taq Gold (AB). The amplification was performed in a 9700-thermal cycler (Applied Biosystems Inc., Foster City, California, 94404, USA), as reported by Lanteri et al. [19]. 

The amplified region was purified and sequenced revealing the bacterial species No other sequences or mixed sequences were obtained in the study. The approximatively 1000 bp obtained sequence was aligned and compared with homologous sequences by WU BLAST software with other high-G+ C-content bacteria. The alignment in GeneBank with the corresponding tract of *Corynebacterium pseudotuberculosis* 16S rRNA gene corresponded to the strain NCTC 3450 (GenBank: X84255.1).

In order to isolate *Corynebacterium pseudotuberculosis* and identify the biovar, two frozen tissue aliquots were sent to the Laboratory of Medical Microbiology and Infectious Diseases of School of Biosciences and Veterinary Medicine, University of Camerino, Italy. Bacteriological culture using Columbia Agar (sheep blood 5%) (Liofilchem^®^, Italy), and Columbia C.N.A.M. Agar (sheep blood 5%) (Liofilchem^®^, Italy) was performed by incubation at 37 °C for 48–72 h in 5–10% of CO_2_ atmosphere (CampyGen^®^, ThermoFisher Scientific, Italy). Phenotypic identification was achieved by biochemical tests for catalase, glucose, sucrose, urea, nitrate reduction, casein, and gelatin hydrolysis [20] using commercial gallery RapID™ CB Plus (Remel, Thermo Fisher Scientific, Italy). According to the manufacturer’s instructions, the reading was performed by the open REMEL software (http://www.remel.com/ERIC/IdentificationSingle.aspx).

Antibiotic susceptibility testing by the Kirby–Bauer disk diffusion method [21] was performed. Kanamycin (30 µg), Amikacin (30 µg), Ampicillin (10 µg), Penicillin G (1 IU), Streptomycin (300 µg), Cefazolin (30 µg), Cefquinome (30 µg), Enrofloxacin (5 µg), Erythromycin (15 µg), Gentamicin (30 µg), Doxycycline (30 µg), Tylosin (30 µg), Rifampin (5 µg), Cotrimazole (23.75 plus 1.25 µg), Azithromycin (15 µg), Cloramphenicol (30 µg), Furazolidone (50 µg), and Oxytetracycline (30 µg) were tested. The zone diameters were interpreted according to guidelines set by the Clinical and Laboratories Standards Institute [22].

**Figure 3:**
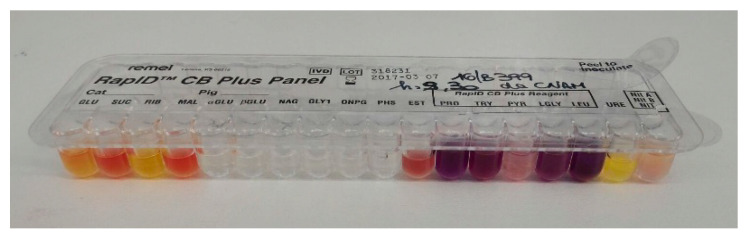
Biochemical gallery (RapID CB Plus, Remel, ThermoFisher, Italy) for strain 1.

By the culture on agar media, two different bacteria were grown. White cream to yellow, circular, aerobic and dry colonies (strain 1), together with deep orange color, circular colonies (strain 2), were recovered. They consisted of Gram-positive, rod-shaped, and catalase positive bacteria. The biochemical identification showed urease test (−), catalase (+), glucose and sucrose (+), gelatin and casein hydrolysis (−), and nitrate reduction (−) for the first microorganism (Figure 3). These results indicated that *Corynebacterium pseudotuberculosis* (Bioscore 1/852 with atypical urease test). In relation to the non-reduction of nitrate to nitrite, biovar Ovis was suspected.

The second strain showed urease test (−), catalase (+), glucose (+), sucrose (−), maltose (−) and leucine-β-naphtylamide (+). By the Remel software reading, the strains resulted *Corynebacterium amycolatum* with atypical maltose and leucine-β-naphtylamide (bioscore 1/272). Antimicrobial drug susceptibility testing showed that both strains were susceptible to Ampicillin (10 µg), Penicillin G (1 IU), Streptomycin (300 µg), Cefazolin (30 µg), Cefquinome (30 µg), Enrofloxacin (5 µg), Rifampicin (5 µg), and Cotrimazole (23.75 plus 1.25 µg). This strain of *Corynebacterium pseudotuberculosis* demonstrated resistance to Kanamycin (30 µg), Amikacin (30 µg), Erythromycin (15 µg), Gentamicin (30 µg), Doxycycline (30 µg), and Tylosin (30 µg), while *Corynebacterium amycolatum* showed resistance only to Tetracyclines.

On the basis of these results, a third cycle of antibiotic therapy with Rifampin (5 mg/kg) twice-a-day orally for three weeks, was administered. A total disappearance of smaller nodules and reduction in size of the larger masses were observed along with a general recovery and improvement of body conditions of the mare. Eight months following treatment, the nodules while smaller were still present and the mare’s clinical condition deteriorated: anorexia, lethargy, weight loss, dehydration, and hyperthermia. Repeat bacteriological culture of three excised tissues were sampled and send to the Laboratory of Medical Microbiology and Infectious Diseases of School of Biosciences and Veterinary Medicine, University of Camerino (Italy). Columbia Agar (sheep blood 5%) (Liofilchem^®^, Italy), and Columbia C.N.A.M. Agar (sheep blood 5%) (Liofilchem^®^, Italy), incubated at 37 °C for 48–72 h in 5–10% of CO_2_ atmosphere (CampyGen^®^, ThermoFisher Scientific, Italy), were used for colony isolation. Two strains grew and antibiotic susceptibility testing by the Kirby–Bauer disk diffusion method showed the susceptibility of both colonies to Ampicillin (10 µg), Penicillin G (1 IU), Cefazolin (30 µg), Cefquinome (30 µg), Enrofloxacin (5 µg), Rifampicin (5 µg), and Cotrimazole (23.75 plus 1.25 µg). Two aliquots of each bacteria were frozen in Cryobank^®^ (Mast Group, Germany), stored at −80 °C, and sent to the Laboratory of Cellular and Molecular Genetics in Brazil for analysis by Quadruplex PCR and MALDI Biotyper tools. 

Bacterial genomic DNA extraction and PCR identification were performed in accordance with a previously described protocol [23]. Quadruplex PCR reactions were performed with a final volume of 50 µL, containing 20 ng of genomic DNA, 0.25 mM dNTPs, 0.1 unit of Taq DNA polymerase (Life Technologies, Carlsbad, CA, USA), 2 mM MgCl_2,_ enzyme buffer (Life Technologies, Carlsbad, CA, USA), 0.5 μM of each primer oligonucleotide, and water to a final volume of 50 μL. The primers used in this study anneal to the *16s rRNA*, *rpoB*, *narG*, and *pld* gene sequences of *Corynebacterium pseudotuberculosis,* in accordance with Almeida et al. [24]. The amplified products were resolved through electrophoresis on 1.0% (*w*/*v*) agarose gels and stained using ethidium bromide.

**Figure 4:**
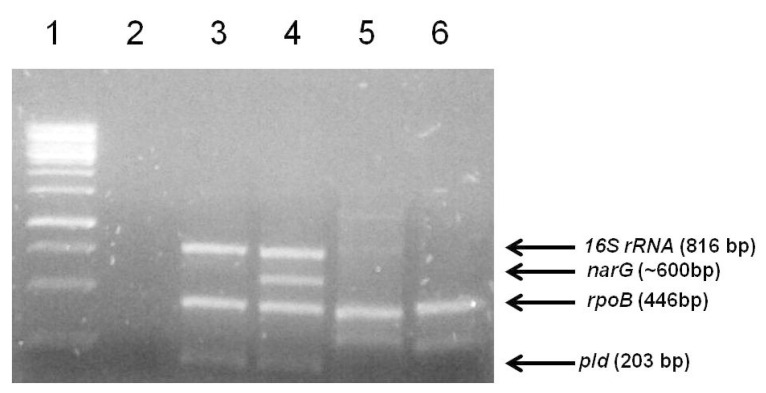
Agarose gel electrophoresis of the quadruplex PCR products for the identification of the two strains isolated from a horse. Lane 1- 1kb Gene Ruler DNA Ladder (Thermo Scientific); Lane 2-Negative Control; Lane 3-*Corynebacterium pseudotuberculosis* biovar Ovis DNA (strain 1002); 4-*Corynebacterium pseudotuberculosis* 258 Equi; 5- DNA from strain 1, isolated from a sick equine; 6-DNA from strain 2, isolated from a sick horse. The arrows indicate the target genes and fragment sizes expected for the *Corynebacterium pseudotuberculosis* amplification profiles.

As expected, the positive control using *Corynebacterium pseudotuberculosis* biovar Equi genomic DNA yielded PCR amplicons corresponding to the 16S rRNA, *rpoB, narG*, and *pld* genes, with 816, 446, ~600, and 203 bp-sized fragments, respectively (Figure 4). In turn, the positive control using *C. pseudotuberculosis* biovar Ovis genomic DNA yielded PCR amplicons corresponding only to the 16S rRNA, *rpoB,* and *pld* genes (Figure 4). As expected, the *pld* gene product was not detected in either of the two strains (Figure 4). For negative control, water was used, and no amplification was detected. The amplification profiles seen for the equine strains 1 and 2 clearly differed from the *Corynebacterium pseudotuberculosis* standard amplification profiles.

Later, a single colony of each bacterial strain was spotted onto a MALDI Biotyper target steel plate using a sterile toothpick. Subsequently, each sample was overlaid with 1 μL matrix consisting of a saturated solution of α-cyano-4-hydroxycinnamic acid (HCCA) (Bruker Daltonics, Bremen, Germany). The preparation was allowed to dry at room temperature. Spectra using the FlexControl MicroFlex LT mass spectrometer (Bruker Daltonics) were acquired in accordance with Assis et al. [25]. Previous calibration of the mass spectrometer was performed using a bacterial standard test [26]. As recommended by the manufacturer, scores ≥2.000 indicated species-level identification, while scores ≥1.700 and <2.000 indicated genus-level identification and scores <1.700 indicated no reliable identification. 

The strain 1 was identified as *Arthrobacter creatinolyticus* (score of 2.387), recently reclassified as *Glutamicibacter creatinolyticus* [27] while the strain 2 was identified as *Dietzia maris* (score of 1.791). As the score for strain 2 was not above the 2.000 threshold, MALDI Biotyper (Bruker Daltonics, Bremen, Germany) indicated reliable identification only at the genus level. Chromosomal sequencing of *Glutamicibacter creatinolyticus* strain was performed using Hiseq technology (Illumina, San Diego, CA, USA), and paired-end libraries (2 × 150 bp). The ab initio assembly was performed by the software Spades, version 3.9. 

The complete genome sequencing of *Glutamicibacter creatinolyticus* LGCM 259 was deposited with the National Center for Biotechnology Information (NCBI) under accession number CP034412; BioProject: PRJNA507728, BioSample: SAMN10502625, Assembly: GCF_006094275.1 and the same became NCBI RefSeq sequence NZ_CP034412.1. On the basis of bacteriological diagnosis, the choice of a final targeted therapy with Trimethoprim and Sulfametoxazole (10 g/100 kg orally the first day, and then 5 g/100 kg for 10 days) resulting in extensive reductions in number and size of the detected lesions and the mare making significant progress. One year following therapy, the mare was markedly improved and pregnant. 

## 3. Discussion

This report describes the clinical-pathological history of a QH mare, lasting not less than two years, characterized by several subcutaneous/muscle lesions treated with different antibiotic therapies that resulted in a decrease in number, but never in the total disappearance. All data resulted in a diffuse granulomatous myositis due to multiple bacteria represented by *Corynebacterium pseudotuberculosis, Corynebacterium amycolatum, Glutamicibacter creatinoltycus,* and bacteria of the genus *Dietzia*. 

*Corynebacterium pseudotuberculosis* is a microorganism facultative intracellular pathogen that exhibits pleomorphic forms, such as coccoids and rods, non-sporulating, non-capsulated, non-motile bacterium, and facultative anaerobe that causes granulomatous and necrotic lesions. In horses, the infection occurs through contact with contaminated soil via skin abrasions or mucous membranes and recent studies support the role of insect vectors, and the incubation period may vary from weeks to months [28]. *Corynebacterium pseudotuberculosis* can be identified through PCR assay with 16S RNA and classified into two biovars based on their ability to reduce nitrate to nitrite [29,30]. Nitrate-negative strains are grouped into the biovar Ovis and they are responsible for Caseous Lymphadenitis (CL), in small ruminants [31] and mastitis in dairy cattle [32]; while the nitrate-positive strains are grouped into the biovar Equi that is responsible of ulcerative lymphangitis, external abscesses and abscesses in internal organs of equines [11,33], and oedematous skin disease in buffaloes [13].

Furthermore, *Corynebacterium pseudotuberculosis* biovar Equi infection in horses is commonly known as “Pigeon Fever” because it leads to the formation of external abscesses on the pectoral muscle region, making it expand, similar to a pigeon breast. Pigeon Fever was described for the first time in California, and since then it has also spread enzootic in some regions of the western United States, which have a low annual precipitation, such as Arizona and Western Texas [33,34]. In fact, the geoclimatic characteristics, e.g., high surface land temperature (>35 °C), dry soil, and areas with low rainfall, appear to strongly influence the appearance of disease outbreaks and could trigger infections in new areas [35]. It cannot be excluded that the similar eco-climatological conditions in southern Italy contribute in the evolution of this disease in the mare reared in Calabria.

Other forms of Infection caused by *Corynebacterium pseudotuberculosis* in horses include abscess formation in the internal organs, that can be fatal if untreated, or ulcerative lymphangitis, which is characterized by the infection of peripheral lymph vessels, severe swelling of the limbs and lameness [36]. External abscesses may occur in a variety of locations including deep intramuscular, axillary, inguinal, and mammary [31].

In the horse in this report, the lesion sites, size, and macroscopic appearance seemed to overlap the typical lesions of *Corynebacterium pseudotuberculosis* infection, although the characterization of the biovar Ovis, together with the absence of a real abscess with purulent material, was not consistent with reports of *Corynebacteirum pseudotuberculosis* in horses. In the horse of this report, the gross appearance of lesions was characterized by a necrotic caseous material surrounded by a fibrotic capsule with small purulent-like foci attributed that may be spread via infection in the lymphatic system.

As recurrence or persistence of *Corynebacterium pseudotuberculosis* infection for more than one year is uncommon [36] co-infection from other bacteria likely contributed to the chronicity of infection in this horse [37,38].

*Dietzia* spp. represents a group of Gram-positive non-spore-forming cocci growing occasionally as short rods, with aerobic metabolism [39]. The genus *Dietzia* was previously classified as *Rhodococcus* and is closely related to the *Actinomycetes*, among which *Corynebacterium* spp., and it is easily and rapidly characterized by using MALDI spiral-TOF MS thanks to the presence of mycolic acids in the cell wall that act similarly to the outer membranes of Gram-negative bacteria [40]. 

*Arthrobacter* species, which belong to the heterogeneous group of coryneform bacteria, consist in Gram-positive, catalase-positive, aerobic, non-spore-forming bacteria and, presently, the genus contains two groups of species referred to as the *Arthrobacter globiformis/Arthrobacter citreus* group and *Arthrobacter nicotianae* group that differs in their peptidoglycan structure [41,42,43]. Infection due to *Arthrobacter* species might have been underestimated because a correct identification of *A. creatinolyticus*, recently reclassified as *Glutamicibacter creatinolyticus* [27,44], is only possible by applying further identification methods (i.e., 16S rRNA gene sequencing or MALDI-TOF MS). Vargha et al. [45] reported that sixteen *Arthrobacter* species could be identified by MALDI-TOF MS.

Infection by *Arthrobacter* spp. is a zoonosis. Reports regarding the isolation of *Arthrobacter* species [42] from human clinical specimens (i.e., wound, urine, or blood) have increased since the late 1990s [46,47]. In veterinary literature, few species belonging to this genus have been isolated, namely *Arthrobacter rhombi* from fish [48], *Arthrobacter nasiphocae* from the nasal cavities of a phoca vitulina [49], *Arthrobacter gandavensis* from cattle [50], and *Arthrobacter equi* from the genital system of a horse [41].

## 4. Conclusions

This case presentation appears to be extremely rare and is of interest due to the etiologic agents found and the peculiar pathological lesions. The isolation of nitrate negative *Corynebacterium pseudotuberculosis* (seen in biovar Ovis) in horses is extremely rare. This appears to be the first report of co-infection due to nitrate negative *C. pseudotuberculosis* combined with *Corynebacterim amycolatum*, *Glutamicibacter creatinolyticus,* and *Dietzia* spp. causing an atypical multibacterial granulomatous myositis in a horse in Italy.

## Accession Number

The complete genome sequencing of *G. creatinolyticus* LGCM 259 was deposited with the National Center for Biotechnology Information (NCBI) under accession number CP034412; BioProject: PRJNA507728, BioSample: SAMN10502625, Assembly: GCF_006094275.1 and the same became NCBI RefSeq sequence NZ_CP034412.1. https://www.ncbi.nlm.nih.gov/nuccore/1679400616.

## Abbreviations

DNA	Deoxyribonucleic acid
CL	Caseous lymphadenitis
CMNR	*Corynebacterium*, *Mycobacterium*, *Nocardia*, and *Rhodococcus* group
C.N.A.M.	Colistin nalidixic acid modified
HCCA	α-cyano-4-hydroxycinnamic acid
HE	Hematoxylin-eosin
PCR	Polymerase chain reaction
IZS	Experimental Zooprophylactic Institute
IU	International unit
MALDI-TOF MS	Matrix-assisted laser desorption/ionization time-of-flight Mass Spectrometry
PAS	Periodic acid—Schiff
QH	Quarter Horse
rRNA	Ribosomal ribonucleic Acid
